# High-grain diet-induced ruminal acidosis triggers systemic inflammation and serum metabolic reprogramming in dairy cows

**DOI:** 10.3389/fvets.2026.1775279

**Published:** 2026-03-02

**Authors:** Bin Zhang, Jin Xie, Xueqiang Li

**Affiliations:** 1 College of Veterinary Medicine, Inner Mongolia Agricultural University, Hohhot, China; 2 College of Animal Science, Shanxi Agricultural University, Taigu, China; 3 College of Veterinary Medicine, Gansu Agricultural University, Lanzhou, China

**Keywords:** high-grain diet, Holstein dairy cow, rumen acidosis, serum metabolomics, systemic inflammation

## Abstract

High-grain diets are widely used to meet the energy demands of high-producing dairy cows; however, excessive grain intake predisposes cows to ruminal acidosis and subsequent systemic inflammation, compromising health and productivity. This study aimed to characterize systemic inflammatory responses and associated serum metabolic alterations induced by high-grain feeding. Fourteen mid-lactation dairy cows fitted with permanent rumen fistulas were subjected to a gradual increase in dietary corn grain to induce ruminal acidosis. Blood samples were collected under normal and acidosis conditions; inflammatory biomarkers were quantified using enzyme-linked immunosorbent assay (ELISA), and serum metabolomic profiles were analyzed by gas chromatography–time of flight mass spectrometry **(**GC-TOF/MS). Rumen pH decreased significantly with increasing dietary corn grain (*p* < 0.05), confirming the successful induction of ruminal acidosis. Serum lipopolysaccharide (LPS) concentrations exhibited an inverted bell-shaped pattern during the induction process (*p* < 0.05), while concentrations of acute-phase proteins, including serum amyloid A (SAA), C-reactive protein (CRP), and haptoglobin (Hp), increased markedly (*p* < 0.05), indicating the development of systemic inflammation. Metabolomic analysis revealed a clear separation between normal and acidosis states, with 37 metabolites significantly different between them. Correlation analysis showed that multiple serum metabolites were closely associated with ruminal pH and inflammatory indicators, particularly SAA, CRP, and Hp. Lipid- and amino acid-related metabolites were positively correlated with inflammatory parameters, whereas several organic acids were negatively correlated, suggesting coordinated metabolic reprogramming during high-grain–induced inflammation. Biomarker analysis identified D-glycerol-1-phosphate and 4-hydroxypyridine as potential serum biomarkers that discriminate acidosis-associated metabolic alterations. In conclusion, a gradual increase in dietary corn grain induces progressive systemic inflammation and pronounced disturbances in serum metabolic homeostasis in lactating dairy cows. These findings highlight the tight linkage between rumen dysfunction and host metabolic regulation and provide potential blood-based biomarkers for early detection of inflammation associated with high-grain feeding.

## 1 Introduction

Sufficient energy and protein supply contribute to higher milk yield and lactation persistence; thus, large amounts of grain are supplied to high-yielding dairy cows to meet energy requirements (1). Cows are transferred from a primarily forage and minimal concentrate diet to a high-degradable concentrate diet shortly after the perinatal period to meet increased energy demands (2). Such high-grain diets are rapidly degraded in the rumen, reducing pH and resulting in subacute ruminal acidosis (SARA) or even acute acidosis (3). The SARA or acute acidosis-affected animals may develop laminitis (4) and intermittent diarrhea, which are detrimental to cow health. Moreover, low pH in the rumen increases the lysis of gram-negative bacteria, leading to the release of lipopolysaccharide (LPS) into the rumen fluid and triggering ruminal inflammation (5). A deteriorated condition will be observed if LPS is released from the rumen into the bloodstream, where it stimulates the production of multiple proinflammatory cytokines and affects the whole metabolic inflammatory response (6). Thus, acute diet transition during early lactation is detrimental to dairy cow health and subsequently reduces milk production.

Blood parameters are considered comprehensive indicators of the body’s metabolic condition, and rumen metabolic disorders induced by a high-grain diet can be reflected by changes in blood metabolites (7). Blood acute-phase proteins, including haptoglobin (Hp), serum amyloid-A (SAA), and C-reactive protein, are considered markers of the body’s inflammatory response (8), and SARA induction initiates the acute-phase response in cattle (9). However, inflammation during the diet transition period is much more systemic, and a more in-depth, meticulous examination should be conducted to elucidate the mechanisms of systemic inflammation during this period. To our knowledge, few studies have explored the metabolic mechanism of this inflammatory status.

Metabonomics is a new system biology method to gain insight into the body’s cellular and physiological response mechanisms, and blood metabolites could be considered as physiological markers to investigate dysfunction (10). Wu et al. (11) elucidated the metabolic characteristics of higher and lower milk protein yield cows based on serum metabolome profiles, and detected potential biomarkers relating to the milk protein yield. Clemmons et al. found that serum metabolites contributed to understand nutrients utilization and feed efficiency of black Angus steers (12, 13). A large number of studies have confirmed that SARA, commonly induced by a high-grain diet, significantly decreases milk yield and impairs overall body health (14). However, the progression and mechanisms of body inflammation, followed by low pH, remain unclear. This study is conducted to reveal the process and mechanism of systemic inflammation during the diet transition period at the metabolic level, discover potential biomarkers to identify systemic inflammation, and alert to rumen acute acidosis in the early lactation period.

## 2 Materials and methods

### 2.1 Animals, diets, and experimental design

Fourteen mid-lactation Holstein dairy cows (day in milk: 182 ± 15 days, milk yield: 18.92 ± 1.43 kg) with permanent rumen fistulas were applied to induce ruminal acidosis by gradually enhancing corn grain content during a 16-day experimental period. An increasing proportion of cracked corn was added to basal diets (Table 1) every 2 days until rumen pH was under 5.8, which is the common sense of acidosis (15, 16), then returned to the basal diet. Ruminal pH was measured 1 h before morning feeding by sampling through the rumen fistulas every 2 days. The first 2 days were considered normal status, and the acidosis status was confirmed when the pH was lower than 5.8. The cows were fed three times daily at 6:30, 14:00, and 19:00, and milked three times. The basal diet for dairy cows was formulated according to the Nutrition Research Council (NRC, 2001) (17) standards, and the dietary composition and nutrient levels are shown in Table 1.

**Table 1:** Composition and nutrient levels of basal diets [%, dry matter (DM) basis].

Ingredients	Content
Com straw silage	27.06
Alfalfa	15.87
Flaked corn	9.78
Cottonseed	6.47
Corn starch	12.55
Brewery mash	8.46
Beet granules	4.12
Soybean meal	9.95
Extruded soybean	0.86
Wheat bran	2.08
NaHCO_3_	0.43
Palm fat powder	1.08
Premix^a^	1.29
Total	100.00
Nutrient levels	
DM	53.9
Crude protein (CP)	17.3
Acid detergent fiber (ADF)	19.8
Neutral detergent fiber (NDF)	30.1
Ca	0.72
P	0.37

a The premix provided the following per kilogram of diet: vitamin A, 7,000 IU; vitamin D₃, 3,000 IU; vitamin E, 50 IU; Cu (as copper sulfate), 10 mg; Zn (as zinc sulfate), 50 mg; Fe (as ferrous sulfate), 70 mg; I (as potassium iodide), 1 mg; Mn (as manganese sulfate), 13 mg; Co (as cobalt chloride hexahydrate), 0.3 mg; and Se (as sodium selenite), 0.15 mg.

All animal experimental protocols were reviewed and approved by the Animal Welfare and Ethics Committee of Inner Mongolia Agricultural University (Approval No. NND2023123) and complied with the national guidelines for the care and use of laboratory animals (SYXK 2022-0031).

### 2.2 Sample collection and analysis

#### 2.2.1 Collection and measurements of blood samples

After morning feeding, 10 mL of blood was collected from the coccygeal vein of each cow daily from days 0–16. Serum was obtained by centrifugation at 3,000 g and 4 °C for 15 min within 30 min of collection and stored at −80 °C for subsequent analysis of inflammatory biomarkers and metabolites. Serum LPS and acute-phase proteins, including serum amyloid A (SAA), C-reactive protein (CRP), and haptoglobin (Hp), were quantified using enzyme-linked immunosorbent assay (ELISA) kits (Jiangsu Meimian Industrial Co., Ltd., China). The ELISA measurements were performed on individual serum samples from each cow (*n* = 14), with each animal considered a single biological replicate.

Because the serum samples were collected repeatedly from the same cows over 17 consecutive days, the data were analyzed using a repeated-measures mixed model, with cow included as a random effect and day as the repeated factor.

#### 2.2.2 Rumen fluid collection and pH measurement

Rumen fluid samples were collected from animals fitted with rumen fistulas. Sampling was performed 1 h before the morning feeding every 2 days. Approximately 50–100 mL of rumen content was obtained from the ventral sac of the rumen using a sterile syringe through the fistula. The samples were immediately filtered through four layers of cheesecloth to remove large feed particles (18). Ruminal pH was measured immediately after collection using a calibrated digital pH meter (PHS-3C, INESA, Shanghai, China) at room temperature. The pH meter was calibrated before each measurement with standard buffer solutions at pH 4.0, 7.0, and 10.0. All measurements were performed in triplicate to ensure accuracy.

### 2.3 Identification and quantification of serum metabolites by GC-TOF/MS

#### 2.3.1 Compounds identification and quantification

First, 50 μL of serum was mixed with 200 μL of methanol and 5 μL of adonitol (0.5 mg/mL stock in dH2O), and vortexed for 30 s. Second, the mixture was ultrasonicated for 5 min and centrifuged at 12,000 rpm and 4 °C for 15 min; then 180 μL of supernatant was transferred and dried in a vacuum concentrator without heating. Third, the dried extract was sequentially incubated with 30 μL of methoxyamination hydrochloride (20 mg/mL in pyridine) for 30 min at 80 °C and with 40 μL of BSTFA reagent (1% TMCS, v/v) for 1.5 h at 70 °C.

To monitor the stability and reproducibility of the GC-TOF/MS system, pooled quality control (QC) samples were prepared by mixing equal aliquots of all serum samples, and 5 μL of FAMEs (in chloroform) were added before analysis. QC samples were injected at regular intervals throughout the analytical run. All serum samples were analyzed using a GC-TOF/MS system (Agilent 7890B, Agilent Technologies, Santa Clara, CA, USA) coupled with a Pegasus HT time-of-flight mass spectrometer (LECO ChromaTOF PEGASUS HT, LECO Corporation, St. Joseph, MI, USA), containing a DB-5MS capillary column coated with 5% diphenyl cross-linked with 95% dimethylpolysiloxane (30 m × 250 μm inner diameter, 0.25 μm film thickness; J&W Scientific, Folsom, CA, USA). Details are provided in Supplementary materials.

#### 2.3.2 Significantly different metabolites identification and pathway analysis

Chroma TOF 4.3X software and Fiehn Rtx5 database of LECO Corporation were applied to extract raw peaks, filter and calibrate the baselines, align peaks, perform deconvolution, identify metabolites, and integrate peak areas. Both mass spectra and retention index matches were considered in metabolite identification. Peaks detected in <50% of QC samples or with relative standard deviation (RSD) > 30% in QC samples were removed.

The GC/MS data were normalized and imported into SIMCA 14.1 software for multivariate analysis, including principal component analysis (PCA) and orthogonal partial least-squares discriminant analysis (OPLS-DA). To reduce the risk of overfitting, the OPLS-DA model was further validated using permutation tests (*n* = 200), and model performance was assessed using *R*^2^ and *Q*^2^ values.

Significantly different metabolites (SDMs) were defined using a combination of multivariate and univariate criteria: metabolites with variable importance in projection (VIP) > 1.0 in OPLS-DA and *p* < 0.10 in univariate analysis were considered significant. This approach is widely accepted in metabolomics studies to balance statistical rigor with biological relevance (19, 20). The Kyoto Encyclopedia of Genes and Genomes (KEGG) database and the Bovine Metabolome Database (BMDB) were used to annotate SDMs.

#### 2.3.3 Biomarker identification

Biomarker analysis was conducted using the “biomarker analysis” module in MetaboAnalyst 4.0.^1^ First, 158 effectively identified metabolites (similarity >400) were imported. Subsequently, data preprocessing, including sum normalization, log transformation, and autoscaling, was performed. Metabolites were selected based on receiver operating characteristic (ROC) curve analysis using the random forest algorithm, considering the area under the curve (AUC), *p*-values, and fold changes. Finally, sensitivity, specificity, predicted probabilities, and combined accuracies of the candidate biomarkers were evaluated with AUC values.

### 2.4 Data analysis

Serum biochemical parameters, acute-phase proteins, and rumen pH were analyzed using the PROC MIXED procedure of SAS 9.2 (SAS Institute, Inc., Cary, NC, USA). Given the repeated measurements obtained from the same cows over time (days 0–16), a repeated-measures mixed model was applied. The statistical model was as follows:

Yi=μ+Ti+Dj+(T×D)ij+Ck+eijk

where Yi is the dependent variable; μ is the overall mean; Ti is the fixed effect of treatment (high-grain feeding); Dj is the fixed effect of day; (T × D)ij is the interaction between treatment and day; Ck is the random effect of cow; and eijk is the residual error. Day was specified as the repeated measure within cow, and an appropriate covariance structure [e.g., autoregressive AR(1)] was selected based on the lowest Akaike information criterion. Tukey’s test was used for multiple comparisons when applicable. Differences were considered significant at *p* < 0.05, and trends were discussed at *p* < 0.10. The results are presented as means ± standard error of the mean (SEM), and error bars in all figures represent SEM.

## 3 Results

### 3.1 Ruminal pH and serum parameters

**Figure 1:**
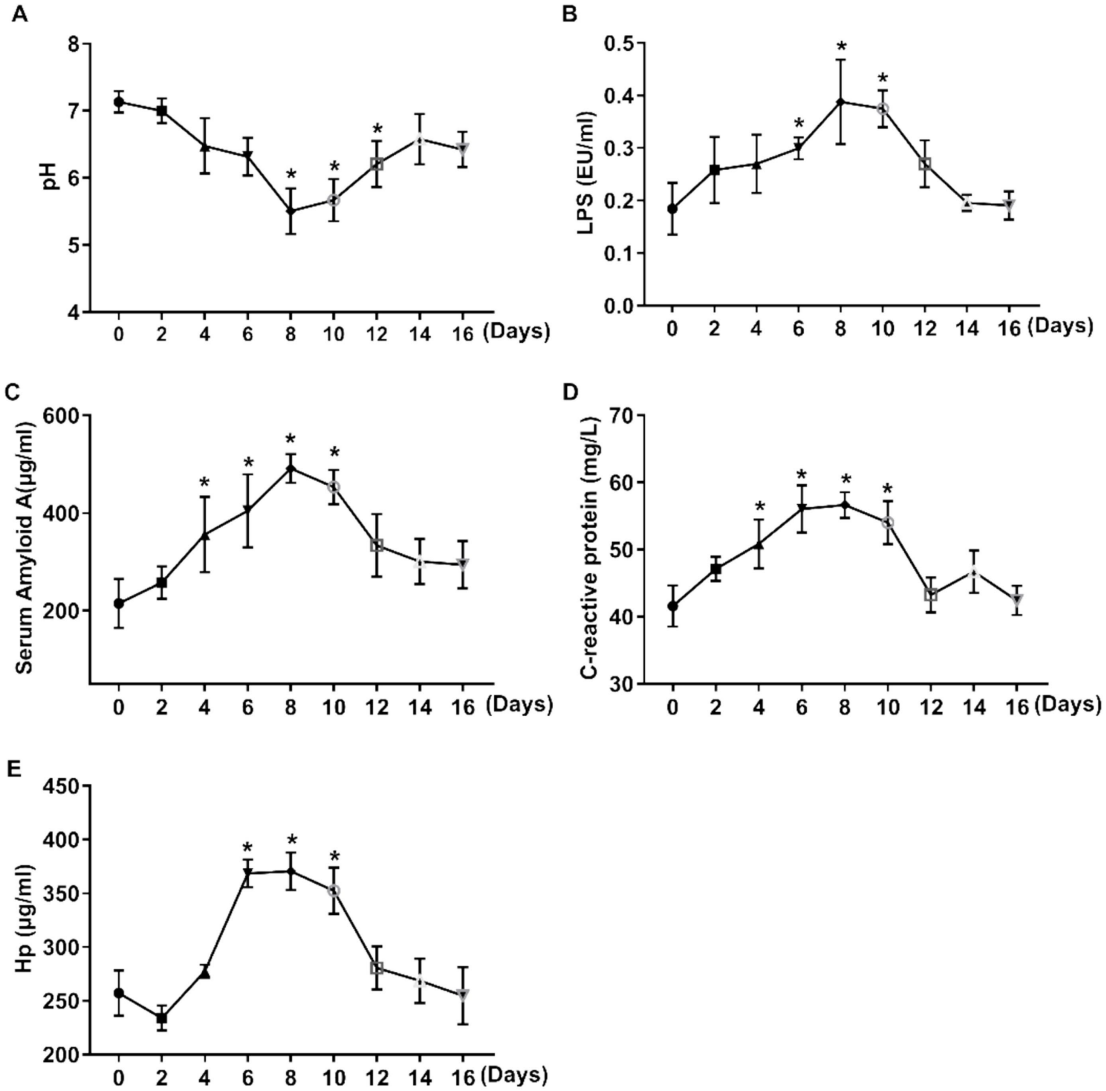
Changes in ruminal pH and serum inflammatory parameters in dairy cows fed increasing dietary corn grain. **(A)** Ruminal pH; **(B)** Serum lipopolysaccharide (LPS); **(C)** Serum amyloid A (SAA); **(D)** Serum C-reactive protein (CRP); and **(E)** Serum haptoglobin (Hp). Data are means ± SEM (*n* = 14 cows). A repeated-measures mixed model was used for statistical analysis, with cow as a random effect. *p < 0.05.

To evaluate the effects of high-grain diet–induced ruminal acidosis on ruminal pH and systemic inflammatory responses, changes in ruminal pH, serum lipopolysaccharide (LPS), and acute-phase proteins were monitored throughout the experimental period. The results showed that ruminal pH decreased significantly (*p* < 0.01) with the enhancement of cracked corn in the TMR diet, but was markedly alleviated after cessation of the inducing procedure (Figure 1A). The serum LPS concentration exhibited an inverted bell-shaped pattern during the whole procedure, with the highest level observed on day 8 (*p* < 0.01) (Figure 1B). Acute-phase proteins, including serum amyloid A (SAA) (Figure 1C), C-reactive protein (Figure 1D), and haptoglobin (Hp) (Figure 1E), increased significantly (*p* < 0.01) with increasing corn grain in the diet and remained at relatively low concentrations for several days after returning to the regular diet.

These results indicate that increasing dietary corn grain markedly reduced ruminal pH and was accompanied by transient elevations in circulating LPS and acute-phase proteins, whereas withdrawal of the inducing diet partially restored ruminal pH and serum inflammatory markers.

### 3.2 Serum metabolite

To characterize the global alterations in serum metabolite profiles between normal and acidosis states, an untargeted metabolomic analysis was performed using GC-TOF/MS. A total of 366 effective peaks were detected in the serum samples after quality control and identification based on the LECO-Fiehn Rtx5 database, including 169 identified metabolites and 197 compounds labeled as “analyte” or “unknown.” The detailed information on effective peaks and compound identification is provided in the Supplementary Table S1.

**Figure 2:**
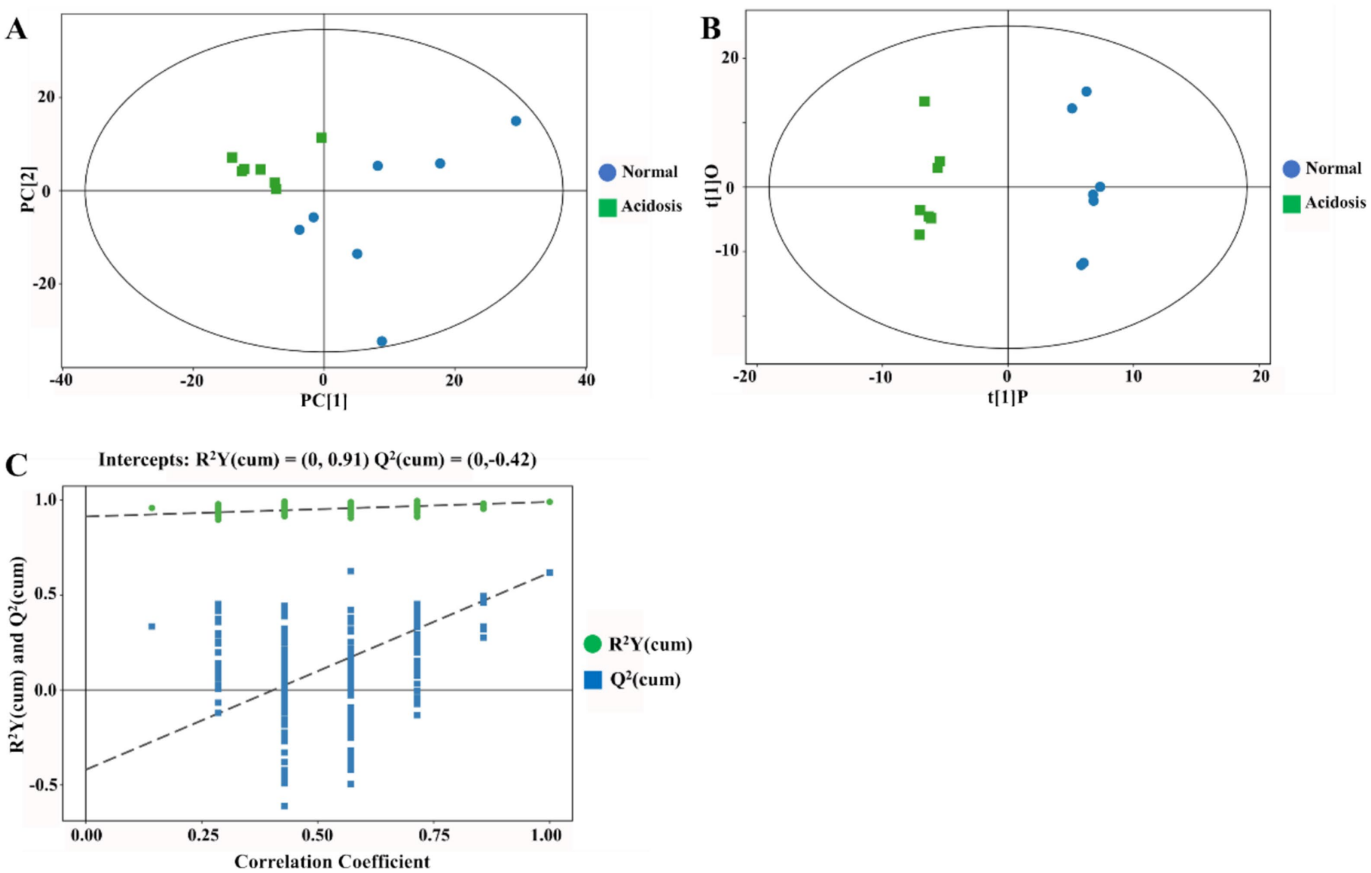
Multivariate analysis of serum metabolomic profiles in dairy cows under normal and acidosis conditions. **(A)** Principal component analysis (PCA) score plot. **(B)** Orthogonal partial least squares discriminant analysis (OPLS-DA) score plot. **(C)** Permutation test for OPLS-DA model validation (*n* = 200). *n* = 7 cows. Green squares indicate acidosis status, and blue circles indicate normal status.

Principal component analysis (PCA) was subsequently conducted to compare serum metabolite profiles between normal and acidosis states (Figure 2A). The PCA score plot showed a clear separation between the two states, with all samples falling within the 95% Hotelling’s T-squared ellipse (Figure 2A). Furthermore, the OPLS-DA score plot showed distinct clustering between normal and acidosis states, with model parameters R^2^Y = 0.99 and *Q*^2^ = 0.618. Permutation tests (*n* = 200) indicated that the OPLS-DA model was robust and suitable for subsequent analyses (Figures 2B,C).

Overall, these results indicate that serum metabolic profiles differed markedly between normal and acidosis states, providing a reliable basis for further identification of differential metabolites.

### 3.3 Significantly different metabolites (SDMs)

**Figure 3:**
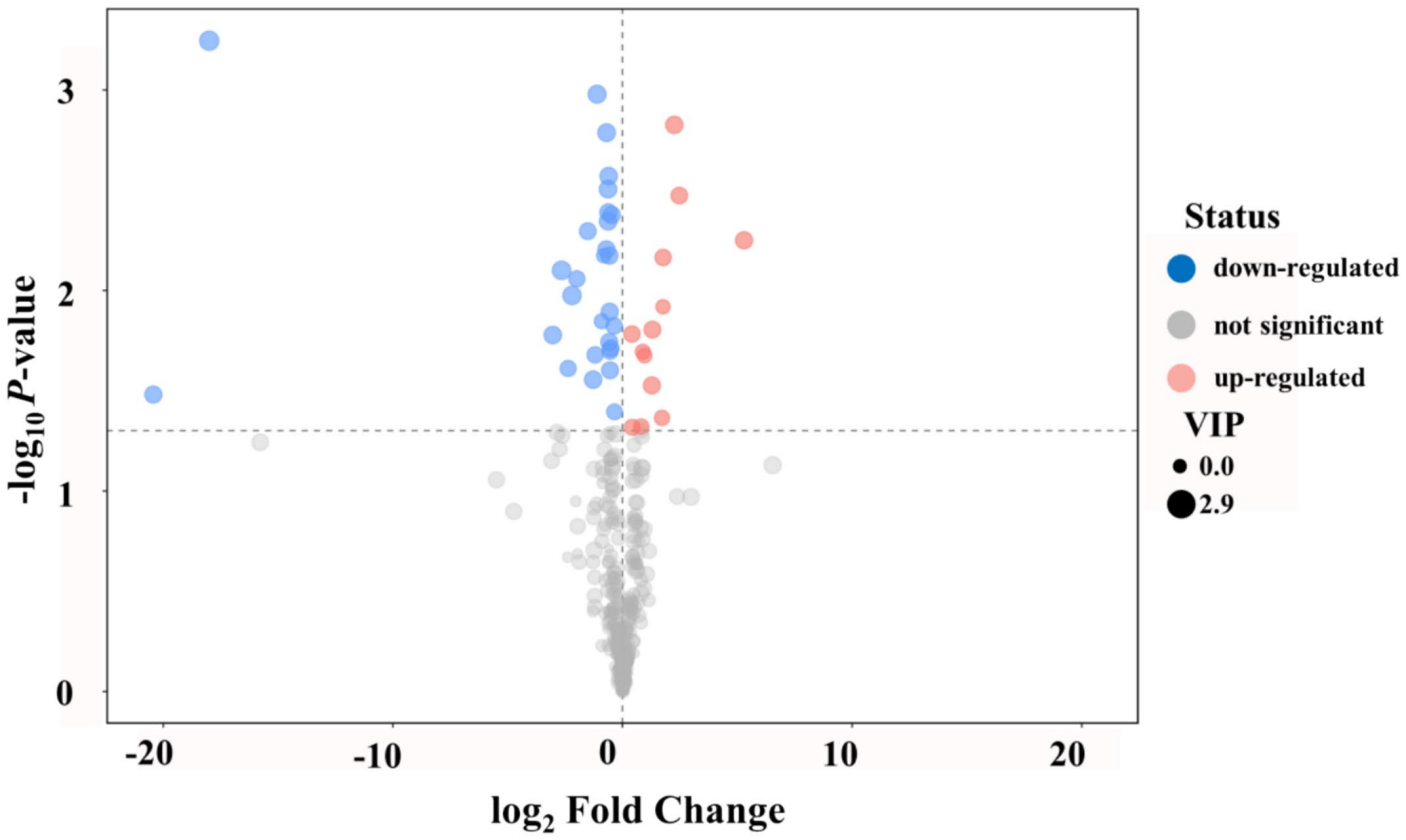
Volcano plot of serum metabolites comparing normal and acidosis states in dairy cows. Red dots represent significantly different metabolites (SDMs) enriched in acidosis, and blue dots represent SDMs enriched in normal status. Dot size indicates variable importance in projection (VIP) values from OPLS-DA analysis. *n* = 7 cows, and SDMs were selected based on VIP > 1.0 and *p* < 0.10. SDM, significantly different metabolite.

To identify the serum metabolites that were most significantly altered between normal and acidosis states, differential metabolite analysis was performed. The volcano plot revealed 37 significantly different metabolites (SDMs) and 29 significantly different unidentified peaks between normal and acidosis states (Figure 3), with 10 SDMs enriched in acidosis and 27 SDMs enriched in normal status. Among the 10 SDMs enriched in acidosis, four metabolites belonged to amino acids and amino acid conjugates, including L-allothreonine [log2 fold change (FC) = 0.88], serine (log2 FC = 0.81), methionine (log2 FC = 0.45), and O-acetylserine (log2 FC = 5.30); two metabolites belonged to organic acids, including L-malic acid (log2 FC = 0.42) and 4-hydroxybutyrate (log2 FC = 1.72); and one metabolite belonged to carbohydrates and carbohydrate conjugates. The 27 SDMs enriched in normal status mainly consisted of carbohydrates and carbohydrate conjugates, organic acids, nucleosides, and nucleoside conjugates. Detailed information is provided in Supplementary Table S1. These findings indicate a distinct shift in serum metabolite composition between normal and acidosis states, providing a foundation for further functional analysis of the identified SDMs.

**Figure 4:**
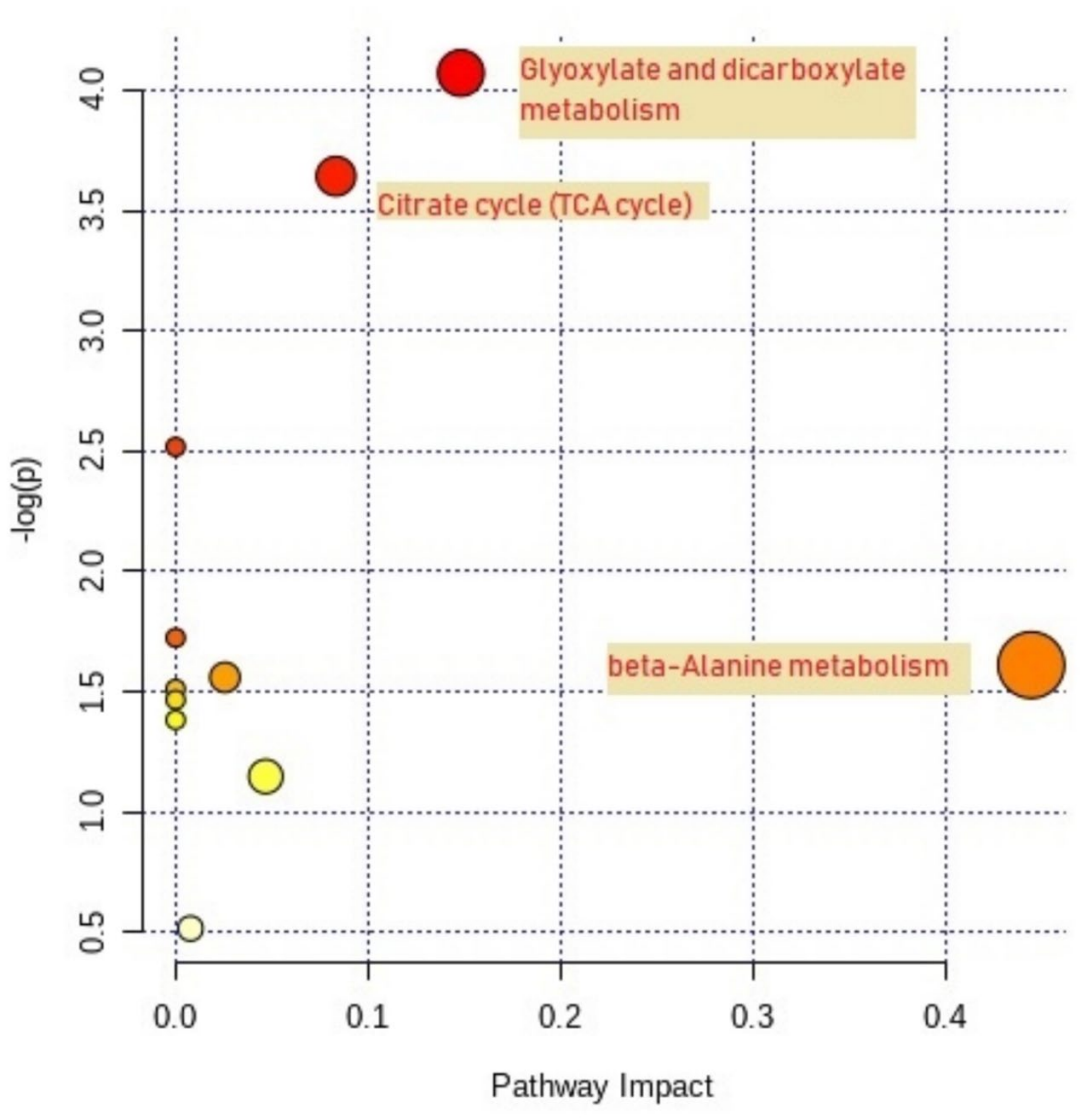
Metabolome view map of significantly different metabolites (SDMs) between normal and acidosis states in dairy cows. Node size indicates pathway impact values, and node color intensity represents pathway enrichment. *n* = 7 cows, and SDMs were identified based on VIP > 1.0 from OPLS-DA and *p* < 0.10. SDM, significantly different metabolite.

Pathway analysis and metabolome view mapping were performed to identify and describe the metabolic pathways of the SDMs (Figure 4). The 37 SDMs were mainly affiliated with Glyoxylate and dicarboxylate metabolism (*p* = 0.02, impact value = 0.15), Citrate cycle (*p* = 0.03, impact value = 0.08), and beta-Alanine metabolism (*p* = 0.20, impact value = 0.44). Glyoxylate and dicarboxylate metabolism and the citrate cycle were characterized as significantly relevant pathways based on *p*-values.

### 3.4 Metabolic biomarkers

**Figure 5:**
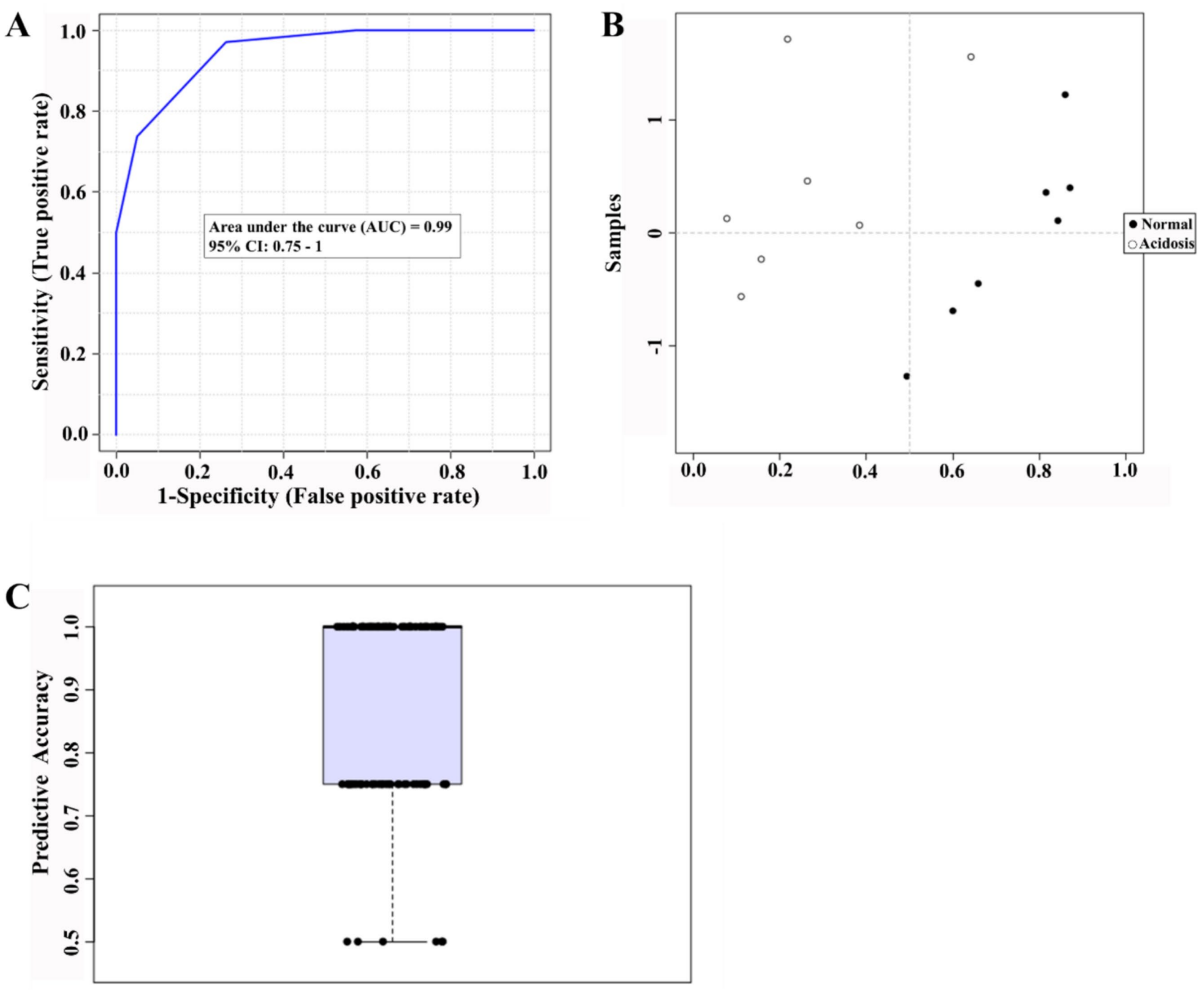
Biomarker analysis of serum metabolite profiles associated with ruminal acidosis in dairy cows. **(A)** Receiver operating characteristic (ROC). **(B)** Probability view plot illustrating. **(C)** Predictive accuracy based on 100 cross-validations. AUC, area under the curve. Data were obtained from *n* = 7 cows.

Biomarker analysis was conducted to identify potential biomarkers to reflect rumen acidosis (Figure 5). Based on 100 cross-validations, 4-hydroxypyridine and D-glycerol 1-phosphate were available to discriminate metabolic differences between normal and acidosis status, with an AUC value and average accuracy of 0.99 and 0.882, respectively; however, an outlier was observed in the probability view image.

### 3.5 Relationships between serum metabolites and blood parameters

**Figure 6:**
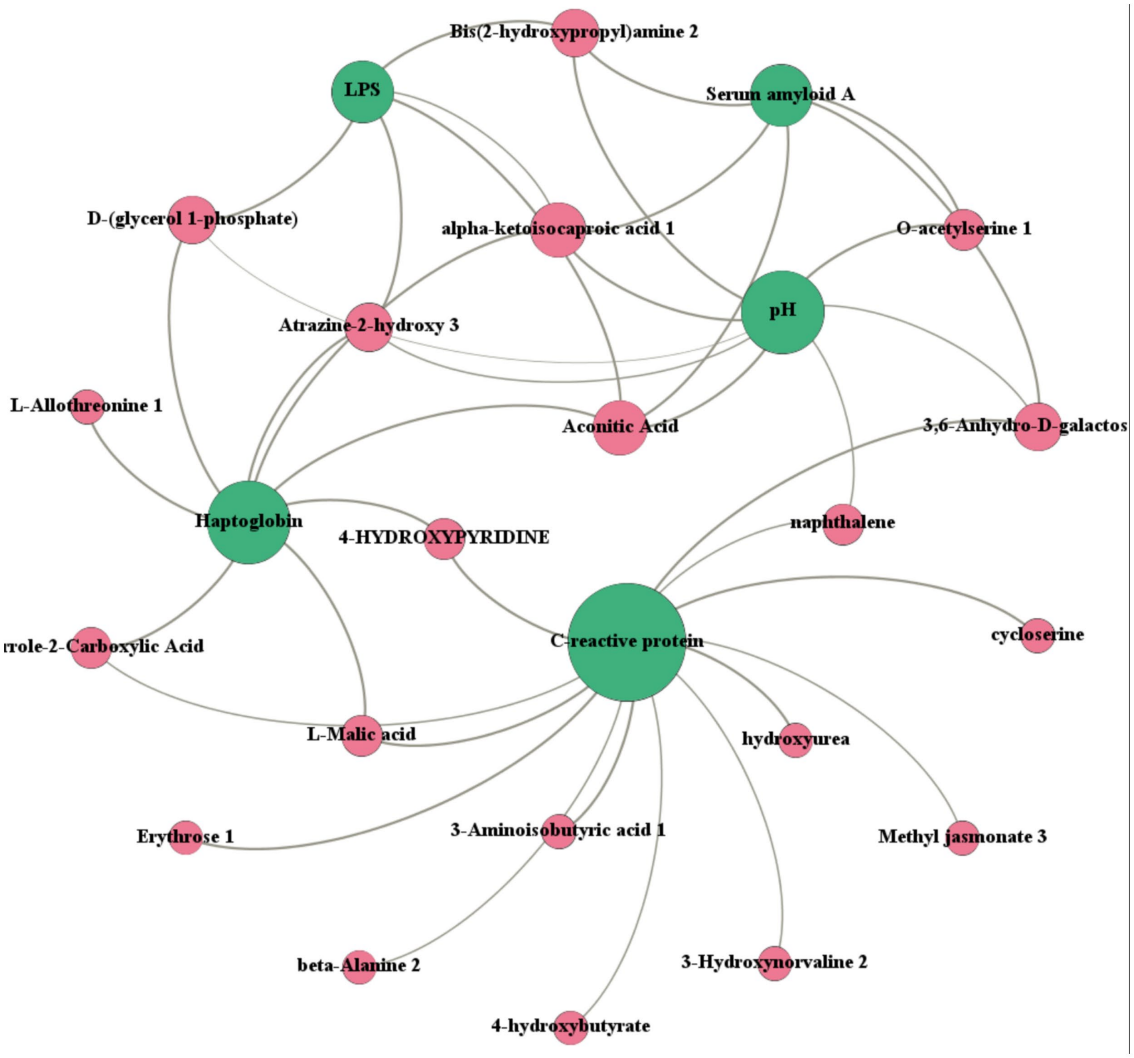
Correlation network between serum metabolites and blood parameters in dairy cows based on Spearman’s correlation coefficients (|*r*| > 0.6, *p* < 0.05). Node size indicates the degree of relevance. Key blood parameters included ruminal pH, serum amyloid A (SAA), C-reactive protein (CRP), and haptoglobin (Hp). *n* = 7 cows.

To explore potential associations between serum metabolites and physiological indicators, correlation analysis was performed between serum metabolites and blood parameters (Figure 6). The resulting network showed that correlations were primarily concentrated on ruminal pH (25 metabolites), SAA (20 metabolites), C-reactive protein (9 metabolites), and haptoglobin (Hp, 5 metabolites). These blood parameters were mainly positively correlated (*R*^2^ > 0.60) with serum lipid metabolites (D-(glycerol 1-phosphate), 4-hydroxypyridine, methyl jasmonate) and amino acid metabolites (aminoisobutyric acid, *β*-alanine), but negatively correlated (*R*^2^ < −0.60) with organic acid metabolites (*α*-ketoisocaproic acid, L-malic acid).

Overall, these results indicate that specific serum metabolites are closely associated with key blood parameters, suggesting coordinated metabolic and inflammatory responses under acidosis conditions.

## 4 Discussion

Ruminal acidosis is a common digestive and metabolic disorder in high-yielding dairy cows (21). Rapid fermentation of high-grain diets leads to a decline in ruminal pH, which not only disrupts ruminal microbial homeostasis and fiber digestion but also promotes endotoxin release, thereby inducing systemic inflammatory responses (22). In severe cases, ruminal acidosis can adversely affect cow health and lactation performance (23). To systematically elucidate the systemic inflammatory responses and underlying metabolic mechanisms associated with high-grain diet–induced ruminal acidosis, mid-lactation dairy cows were selected for this study. Acidosis was induced by progressively increasing the proportion of corn in the diet, while serum lipopolysaccharide (LPS), acute-phase proteins [serum amyloid A (SAA), C-reactive protein (CRP), and haptoglobin (Hp)], and serum metabolites were continuously monitored. The results showed that increasing dietary corn levels significantly reduced ruminal pH, accompanied by elevated serum inflammatory markers and pronounced alterations in serum metabolic profiles. These findings indicate that high-grain diets not only disrupt the ruminal environment but also trigger systemic inflammatory responses, and adaptive changes in energy metabolism and amino acid- and lipid-related metabolic pathways (24). Based on these results, the following sections will present a detailed discussion of changes in serum parameters, significantly altered metabolites, and potential biomarkers associated with ruminal acidosis.

### 4.1 Serum parameters

Under ruminal acidosis, the decline in ruminal pH disrupts microbial homeostasis and facilitates the translocation of lipopolysaccharides (LPS) from the gastrointestinal tract into the bloodstream, thereby initiating a systemic inflammatory response (25). In the present study, a gradual increase in the proportion of dietary corn resulted in a significant reduction in ruminal pH, accompanied by pronounced elevations in serum LPS, hemoglobin-binding protein (HbBp), and C-reactive protein (CRP), which changed inversely to ruminal pH. These results demonstrate that serum LPS and acute-phase proteins are sensitive indicators of inflammation triggered by high-grain feeding (26). The increased levels of acute-phase proteins further reflect the onset of systemic inflammation under acute ruminal acidosis, and their dynamic changes are consistent with serum LPS concentrations (27), collectively supporting the conclusion that high-grain diets can induce systemic inflammatory responses.

These alterations in serum parameters not only illustrate the direct influence of ruminal acidosis on the gastrointestinal environment but also demonstrate the activation of host immune defense mechanisms during the acidosis state. The elevation of serum acute-phase proteins may contribute to the modulation of inflammatory signaling pathways, functioning as a protective response against endotoxin challenge (28). Meanwhile, the translocation of LPS into the bloodstream activates multiple inflammation-associated pathways, promoting the production of pro-inflammatory mediators and ultimately establishing a typical systemic inflammatory profile. This observation is consistent with previous reports showing that high-grain feeding or ruminal acidosis markedly increases serum LPS and acute-phase protein levels in dairy cows, supporting their potential use as sensitive indicators for monitoring inflammatory status and assessing health conditions (29).

In conclusion, this study clearly characterizes the inflammatory features induced by high-grain diet–related ruminal acidosis through dynamic changes in serum parameter, providing a foundation for subsequent investigation of serum metabolic alterations and metabolic regulatory mechanisms in the host.

### 4.2 Significantly different metabolites

The SDM analysis indicated that four of the seven amino acids and their conjugates were enriched in the acidosis status. L-allothreonine is down-stream product of Glycine (Gly), which is an important gluconeogenic amino acids (30, 31), thus the enriched L-allothreonine in acidosis status suggested that more Gly were decayed to supply energy; in contrast, Gly plays an important role in oxygen stress, cell membrane injury, tumor metastasis and other processes (32, 33), thus the enhanced L-allothreonine in acidosis status explained the systemic inflammation. L-allothreonine is also the substrate of serine (Ser) hydroxymethyl transferase, which catalyzes the invertible interaction of Gly and Ser. Thus, the enrichment L-allothreonine and serine in acidotic state indicated that Gly, Ser, and Thr metabolism may be significantly enhanced, which in turn, increased Gly degradation. Although several studies have identified Gly (34), Ser, and Thr metabolism as the key pathway in dairy cows (11, 35), this pathway is not the key pathway in the current study. Majority of the carbohydrates and carbohydrate conjugates and organic acids were enriched in normal status, suggesting that systemic inflammation adjusted carbohydrate metabolism significantly.

Previous studies have found that lipid metabolites played an important role in the inflammatory response during calving (36, 37), myo-inositol is the most predominant form of inositol, which is involved in fat lipolysis; thus, the decreased myo-inositol during the inflammatory process in the current study might interfere with fat metabolism (38). Methyl jasmonate could stimulate reactive oxygen species (ROS) scavenging and have anti-inflammatory ability through inhibiting the Nuclear factor kappa B (NF-κB) pathway (39). Besides, Methyl jasmonate has been studied as an anti-inflammatory agent due to its ability to alleviate the acute and chronic inflammatory conditions (40, 41). Thus, the downregulation of serum methyl jasmonate suggests that the anti-inflammatory capacity was attenuated by a high-grain diet. The glycolysis procedure led to an increase in lactic acid in dairy cows (42); thus, the decreased lactate under low pH suggested a lower glycolytic activity and suppressed glucose catabolism process to supply energy. As the pre-step, the dramatically altered glycolysis activity induced adjustments to the citrate cycle pathway. Therefore, systemic inflammation resulting from an acute transition to a high-grain diet significantly altered energy metabolism and supply. In contrast, atrazine is a ubiquitous herbicide, which is applied to control weeds in grass farma. The decreased serum atrazine-2-hydroxy in the current study may be explained by the less grass feeding (43).

**Figure 7:**
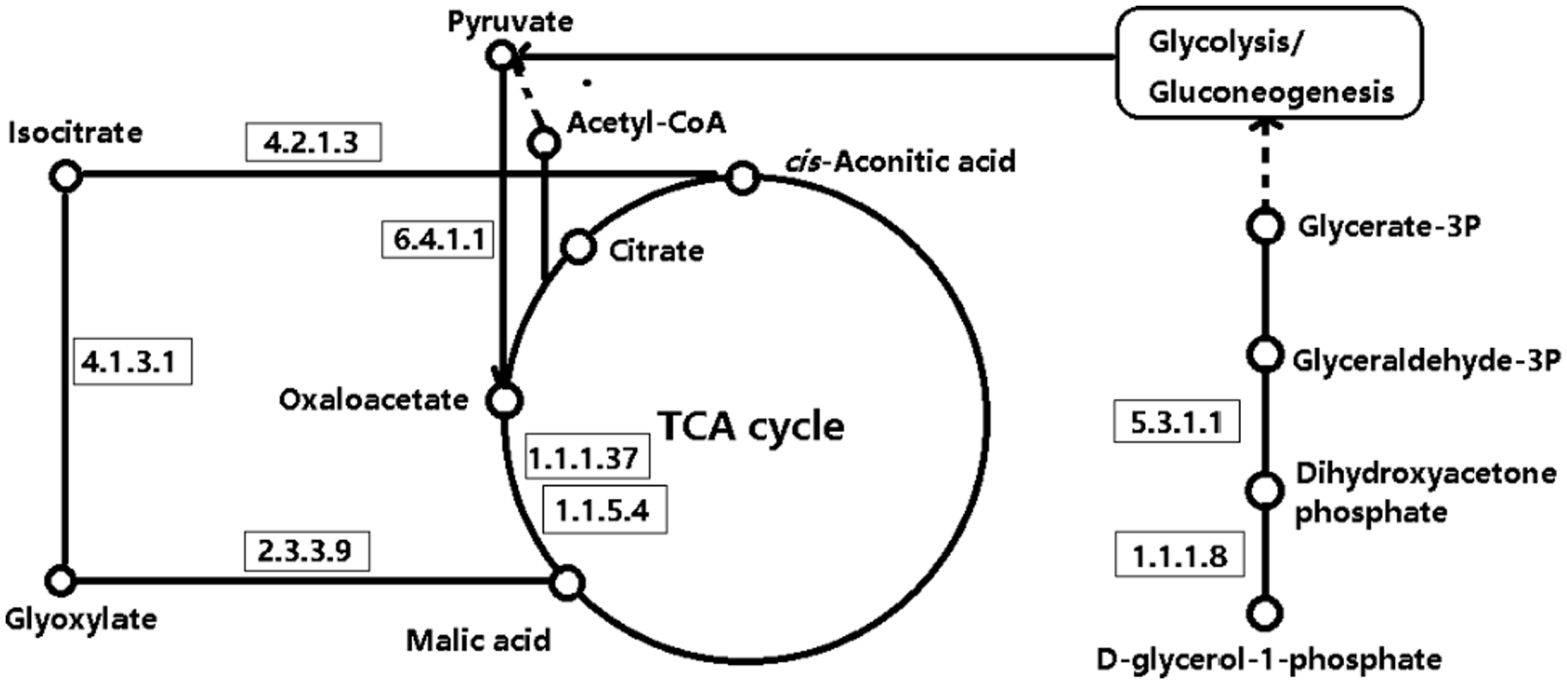
Manually linked Kyoto encyclopedia of genes and genomes (KEGG) pathways showing key serum metabolites in dairy cows under normal and acidosis states. Nodes represent metabolites, and arrows indicate metabolic flux directions. Numbers in boxes correspond to the Enzyme Commission (EC) numbers of the enzymes catalyzing each reaction. TCA, tricarboxylic acid cycle.

**Figure 8:**
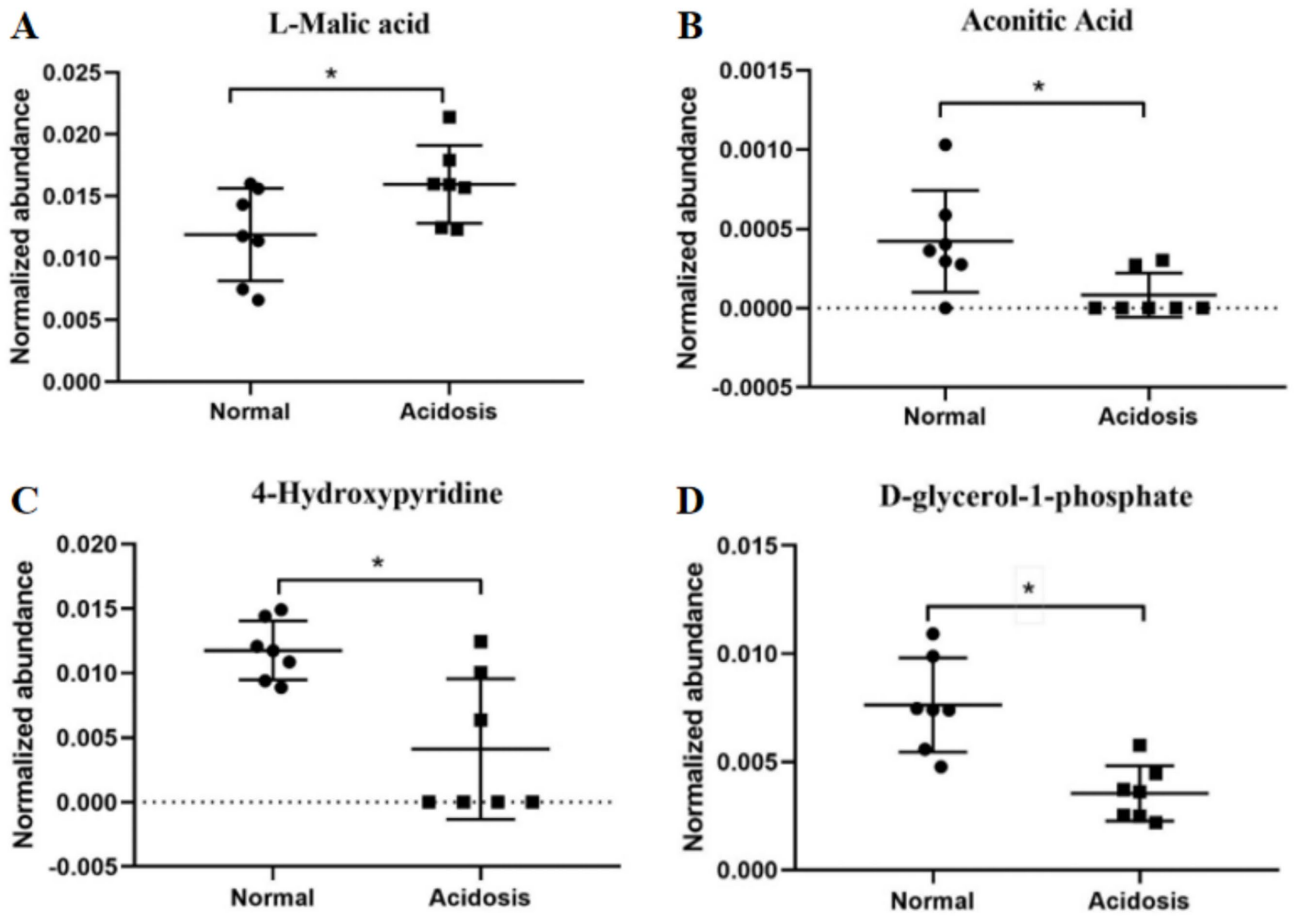
Normalized relative abundance of normal and acidosis status for **(A)** L-malic acid, **(B)** aconitic acid, **(C)** 4-hydroxypyridine, and **(D)** D-glycerol-1-phosphate.

Two integrated key metabolic pathways were manually integrated together in the current study (Figure 7). Glyoxylate and dicarboxylate metabolism and the citrate cycle were identified by L-malic acid [log_2_ (FC) = 0.42] and aconitic acid [log_2_ (FC) = −2.37], implying that the glucose metabolism was significantly involved with systemic inflammation. Sun et al. also found that Glyoxylate and dicarboxylate metabolism and the tricarboxylic acid (TCA) cycle pathway were the functionally enriched pathways in lactating dairy cows (35). Malic acid, which is the key compound in the citrate cycle, was significantly greater in acidosis status (*p* = 0.04, Figure 8A). Malic acid is associated with acetyl-CoA, which is the starting point of the citrate cycle and is key to fat and carbohydrate metabolism (43, 44), and greater malic acid may enhance mitochondrial function and the citrate cycle. Thus, the enrichment of malic acid in serum metabolites suggested greater fat and carbohydrate catabolism. The glyoxylate cycle allows humans to use fats for the synthesis of carbohydrates (45, 46), and Gly and glyoxylate can be converted by glutamate-glyoxylate aminotransferase, the increased downstream compounds of Gly indicated that more Gly was degraded, therefore glyoxylate and dicarboxylate metabolism was enhanced to convert more glyoxylate to Gly (47). Along with the lower aconitic acid (*p* = 0.02, Figure 8B) in acidosis status indicated that glyoxylate and dicarboxylate metabolism contributed to enhancing Citrate cycle intermediates to supply more energy (48). Thomas et al. reported that (S)-malate and cis-aconitate decreased at the peak of mastitis infection, suggesting that the TCA cycle is downregulated in bovine mastitis (49). In the present study, the observed decreases in citric acid and aconitic acid similarly indicate a weakened TCA cycle, suggesting the citrate cycle could be manipulated by regulating glyoxylate and dicarboxylate metabolism to improve energy supply.

### 4.3 Biomarkers

An AUC value greater than 0.8 is considered an excellent predictor for biomarker selection; thus, the AUC in the current study indicated a stable model to set 4-hydroxypyridine and D-glycerol 1-phosphate (sn-glycerol-3-phosphate, G3P) as body inflammatory biomarkers between normal and acidosis status (Figures 8C,D). Glycerol can be hydrolyzed into glycerol-3-phosphate and glyceric acid through glycolysis (7). D-glycerol 1-phosphate is a chemical intermediate in the glycolysis metabolic pathway, and can be converted to dihydroxyacetone phosphate, which could be rearranged to glyceraldehyde-3-phosphate, then fed into glycolysis (50). Lactate, the end-product of glucose metabolism under anaerobic conditions, prevents the inhibition of glycolysis (51). Thus, the decreased serum lactate and D-glycerol 1-phosphate in acidosis status implied an enhanced glycolysis to supply energy through fatty acid dehydrolysis and anaerobic glycolysis. Along with the predominant citrate cycle pathway, the glucose and fat metabolism may be altered significantly during a high-grain diet (52). Although few studies have involved 4-hydroxypyridine, Zhang et al. (53) found that a high-concentration diet induced lower rumen fluid 3-hydroxypyridine, which is the isomer of 4-hydroxypyridine.

It should be noted that both D-glycerol-1-phosphate and 4-hydroxypyridine were identified as biomarkers based on their significantly decreased concentrations in cows experiencing acidosis. Biomarkers characterized by downward trends present particular analytical challenges, as accurate discrimination of low-abundance metabolites from background noise requires high sensitivity and specificity. In the present study, these metabolites were reliably quantified using mass spectrometry-based metabolomic approaches, which remain the gold standard for sensitive detection of small-molecule metabolites. However, such platforms are currently not routinely applicable in on-farm diagnostic settings. At present, these biomarkers may therefore be more suitable for laboratory-based monitoring or research applications. Future translation to practical farm-level diagnostics would likely depend on the development of targeted analytical methods, such as simplified liquid chromatography–mass spectrometry (LC–MS/MS) assays or emerging biosensor technologies, to enable robust and cost-effective detection of low-concentration metabolites under field conditions.

### 4.4 Relationships between serum metabolites and blood parameters

Correlation analysis further revealed the intrinsic link between systemic inflammation and serum metabolic reprogramming during high-grain-induced ruminal acidosis. Network analysis showed that ruminal pH exhibited the highest number of significant correlations with serum metabolites, indicating that changes in the rumen environment represent a key initiating factor driving metabolic and inflammatory responses. As ruminal pH declined, serum acute-phase proteins (SAA, CRP, and Hp) increased and showed significant positive correlations with specific lipid- and amino acid-related metabolites, suggesting that activation of the inflammatory response is closely associated with lipid and amino acid metabolism.

Among these, the positive correlations between lipid-related metabolites—such as D-glycerol-1-phosphate and 4-hydroxypyridine—and inflammatory markers may reflect enhanced fat mobilization and membrane lipid remodeling under inflammatory conditions, providing energy and structural substrates required for immune activation (54). In addition, the observed association between methyl jasmonate—an endogenous metabolite with reported anti-inflammatory and antioxidant activity (41)—and inflammatory markers implies that intrinsic anti-inflammatory regulatory capacity may be either suppressed or reprogrammed during the systemic inflammatory response triggered by ruminal acidosis (55). Amino acid metabolites (such as amino isobutyric acid and *β*-alanine) showed positive correlations with inflammatory markers, suggesting that under the stress induced by a high-grain diet, the body may enhance amino acid catabolism and conversion to provide substrates required for immune activation and tissue repair (56). In contrast, organic acid metabolites (including *α*-ketoisovaleric acid and L-malic acid) showed significant negative correlations with inflammatory indicators, implying potential suppression or redistribution of the tricarboxylic acid (TCA) cycle and associated energy-generating pathways during inflammation. Collectively, this metabolic shift indicates that under acute systemic inflammatory states, the organism may transition from oxidative metabolism towards more stress-adaptive metabolic patterns.

Overall, the correlations between serum metabolites and inflammatory markers indicate that ruminal acidosis induced by a high-grain diet not only triggers a systemic inflammatory response but also involves coordinated regulation of lipid, amino acid, and energy metabolism. These findings provide new mechanistic evidence supporting the interplay between inflammation and metabolic reprogramming in ruminal acidosis and highlight the potential of specific serum metabolites as biomarkers for evaluating systemic inflammatory and metabolic status.

### 4.5 Immunometabolic interpretation of metabolomic alterations induced by high-grain feeding

High-grain–induced ruminal acidosis not only disrupts rumen fermentation but also imposes a systemic immunometabolic challenge. In the present study, the decline in ruminal pH was accompanied by elevated circulating LPS and acute-phase proteins, as well as marked alterations in serum metabolomic profiles, particularly those related to amino acid metabolism. These results indicate that high-grain feeding induces metabolic reprogramming closely linked to inflammatory activation.

Amino acids function not only as metabolic substrates but also as key signaling molecules that regulate cellular metabolism and immune responses through the mechanistic target of rapamycin (mTOR) pathway. mTOR integrates nutrient availability with inflammatory and metabolic signals, thereby coordinating anabolic processes and immune activation (57). The disturbance of amino acid–associated metabolites observed in this study suggests impaired nutrient sensing and altered systemic energy allocation under acidosis conditions. Importantly, mTOR activity is tightly regulated by cellular energy status and interacts with AMP-activated protein kinase (AMPK) to balance anabolic and catabolic pathways during metabolic stress. Under high-grain–induced inflammatory and energy overload, suppression of anabolic metabolism and activation of stress-responsive pathways may occur, thereby contributing to metabolic inefficiency and inflammatory burden. Recent evidence further indicates that nuclear mTOR signaling orchestrates transcriptional programs involved in cellular growth, metabolism, and stress adaptation (58), providing a mechanistic basis for the coordinated metabolic shifts observed in serum. Prolonged activation of such immunometabolic pathways has been associated with impaired growth efficiency and metabolic dysfunction (59).

Collectively, our findings support a model in which high-grain–induced acidosis triggers systemic inflammation and amino acid–centered metabolic reprogramming, mediated in part by mTOR signaling and its interaction with energy stress pathways, thereby linking ruminal disturbances to systemic metabolic alterations.

Although the sample size was sufficient to detect clear differences in ruminal pH, serum biochemical parameters, and inflammatory markers, the relatively limited number of animals may reduce the statistical power of untargeted metabolomic analyses. Accordingly, the metabolomic results should be interpreted with caution and require further confirmation in larger cohorts and through targeted validation.

## 5 Conclusion

This study successfully established a ruminal acidosis model in lactating dairy cows by progressively increasing the proportion of corn in the diet. It systematically clarified the characteristics of systemic inflammation and the serum metabolic regulatory mechanisms associated with high-grain-induced ruminal acidosis. The results demonstrated that increasing grain proportion led to a significant decline in rumen pH, accompanied by markedly elevated serum concentrations of lipopolysaccharide (LPS) and acute-phase proteins (SAA, CRP, and Hp), indicating that disruption of the ruminal environment triggers systemic inflammatory responses.

Serum metabolomic profiling further revealed extensive remodeling of the serum metabolic landscape under acidosis, with a clear distinction between metabolic states in normal and acidosis conditions. Differentially expressed metabolites were primarily enriched in pathways related to lipid metabolism, amino acid metabolism, and energy metabolism, suggesting that systemic inflammation is closely associated with substantial reallocation of metabolic substrates and adjustment of energy supply mechanisms. Correlation analysis also confirmed strong associations among rumen pH, inflammatory indicators, and multiple serum metabolites, highlighting the tight coupling between inflammatory activation and metabolic regulation. Additionally, biomarker screening identified D-glycerol-1-phosphate and 4-hydroxypyridine as potential serum biomarkers that effectively distinguish healthy cows from those experiencing ruminal acidosis, offering promising indicators for early monitoring and diagnosis of systemic inflammation in dairy cows on high-grain diets.

In conclusion, ruminal acidosis induced by high-grain feeding not only disturbs ruminal homeostasis but also provokes systemic inflammation and induces substantial metabolic reprogramming at the serum level. These findings deepen current understanding of inflammatory mechanisms associated with ruminal acidosis from a metabolomic perspective and provide a theoretical basis and potential intervention targets for optimizing dietary strategies in lactating dairy cows to prevent acidosis-related metabolic disorders.

## Data Availability

The original contributions presented in the study are included in the article, and further inquiries can be directed to the corresponding author.

## Footnotes

1
https://www.metaboanalyst.ca/
