# Supplementary material for: High-grain diet-induced ruminal acidosis triggers systemic inflammation and serum metabolic reprogramming in dairy cows
Source: Front Vet Sci. 2026 Mar 2;13:1775279. doi: 10.3389/fvets.2026.1775279 (PMC12989408; doi:10.3389/fvets.2026.1775279)
Supplement: Supplementary file 1 [file Table_1.docx]

**Supplementary data**

This Supplementary Information contains:

1 Identification and quantification of serum metabolite profiles by GC-TOF/MS.

2 Table S1

**Identification and quantification of serum metabolite profiles by GC-TOF/MS.**

***GC-TOF-MS Analysis：***

A 1μL serum sample was injected in split less mode with Helium as carrier gas, a 3 mL min^−1^front inlet purge flow and 1mL min^−1^ gas flow rate was applied to analyze procedure. Kept initial temperature at 50°C for 1min, then raised to 310°C at a rate of 20°C min^−1^ and maintained for 6min. The injection, transfer line, and ion source temperatures were 280, 280, and 250°C, respectively. The energy was -70eV in electron impact mode. The mass spectral data were obtained in full-scan mode at a rate of 12.5 spectra/second within the m/z range of 50-500 after a solvent delay of 4.78min.

**Table S1 Significant different metabolites between normal and acidosis status**

| **Peak** | **Similarity** | **R.T.** | **Mass** | **VIP** | **P** | **Q** | **Log_Fold hange** |
| --- | --- | --- | --- | --- | --- | --- | --- |
| citric acid | 966 | 10.2941,0 | 273 | 1.470 | 0.08 | 0.363 | -0.442 |
| creatine | 960 | 8.8418,0 | 115 | 1.541 | 0.08 | 0.366 | -0.444 |
| myo-inositol | 929 | 11.6911,0 | 217 | 2.088 | 0.03 | 0.241 | -1.279 |
| L-Allothreonine | 926 | 7.69713,0 | 73 | 1.272 | 0.02 | 0.203 | 0.884 |
| serine | 903 | 7.52259,0 | 204 | 1.310 | 0.05 | 0.316 | 0.807 |
| oleic acid | 876 | 12.2978,0 | 98 | 2.880 | 0.00 | 0.127 | -17.996 |
| alpha-ketoisocaproic acid | 849 | 6.49698,0 | 200 | 1.848 | 0.02 | 0.189 | 1.303 |
| methionine | 841 | 8.60987,0 | 176 | 1.349 | 0.09 | 0.385 | 0.454 |
| L-Malic acid | 837 | 8.36087,0 | 147 | 1.535 | 0.05 | 0.311 | 0.424 |
| hydroxyurea | 832 | 6.6564,0 | 277 | 1.898 | 0.02 | 0.197 | -0.577 |
| uric acid | 792 | 11.7025,0 | 441 | 1.997 | 0.00 | 0.132 | -0.617 |
| D-(glycerol 1-phosphate) | 753 | 9.97393,0 | 299 | 2.311 | 0.00 | 0.127 | -1.107 |
| Lactobionic Acid | 683 | 14.4085,0 | 191 | 1.545 | 0.09 | 0.383 | -5.489 |
| 4-HYDROXYPYRIDINE | 663 | 6.17983,0 | 152 | 1.843 | 0.01 | 0.133 | -1.511 |
| Fructose 2,6-biphosphate degr prod | 609 | 12.2255,0 | 211 | 1.270 | 0.09 | 0.380 | -0.539 |
| Diglycerol | 579 | 10.024,0 | 116 | 1.672 | 0.08 | 0.365 | 0.845 |
| 4-hydroxybutyrate | 570 | 6.66193,0 | 131 | 1.316 | 0.04 | 0.297 | 1.722 |
| caprylic acid | 564 | 6.90187,0 | 201 | 1.648 | 0.05 | 0.320 | -0.292 |
| Erythrose | 557 | 8.16307,0 | 73 | 1.965 | 0.01 | 0.176 | -0.556 |
| 3-Aminoisobutyric acid | 532 | 8.2286,0 | 154 | 1.711 | 0.02 | 0.186 | -0.365 |
| Adenosine 5-monophosphate | 530 | 15.9347,0 | 169 | 2.120 | 0.02 | 0.192 | -3.036 |
| 2-Deoxy-D-galactose | 524 | 10.169,0 | 144 | 1.530 | 0.05 | 0.318 | -2.846 |
| Aconitic Acid | 521 | 9.91005,0 | 229 | 1.600 | 0.02 | 0.224 | -2.368 |
| Sophorose | 518 | 14.6803,0 | 166 | 1.379 | 0.05 | 0.310 | 0.815 |
| 3-Hydroxynorvaline | 507 | 7.53407,0 | 221 | 2.115 | 0.00 | 0.132 | -0.627 |
| beta-Alanine | 493 | 8.04961,0 | 174 | 1.467 | 0.04 | 0.288 | -0.350 |
| N-Methyl-L-glutamic acid | 489 | 8.89867,0 | 158 | 1.727 | 0.06 | 0.332 | -15.776 |
| Bis(2-hydroxypropyl) amine | 474 | 7.29847,0 | 160 | 1.884 | 0.03 | 0.227 | -0.540 |
| Pyrrole-2-Carboxylic Acid | 451 | 7.51236,0 | 240 | 1.627 | 0.01 | 0.150 | -1.987 |
| Atrazine-2-hydroxy | 411 | 10.5223,0 | 140 | 2.208 | 0.00 | 0.127 | -0.692 |
| Methyl jasmonate | 398 | 9.67007,0 | 282 | 2.144 | 0.00 | 0.131 | -0.630 |
| 3,6-Anhydro-D-galactose | 389 | 9.9366,0 | 248 | 2.086 | 0.00 | 0.132 | -0.474 |
| salicin | 368 | 13.4483,0 | 156 | 1.706 | 0.01 | 0.133 | 1.770 |
| O-acetylserine | 345 | 7.73354,0 | 174 | 2.003 | 0.01 | 0.133 | 5.295 |
| cycloserine | 295 | 9.39153,0 | 248 | 2.048 | 0.01 | 0.133 | -0.700 |
| 1,2-Cyclohexanedione | 293 | 8.3011,0 | 281 | 1.460 | 0.07 | 0.350 | -0.337 |
| naphthalene | 199 | 6.57215,0 | 57 | 1.974 | 0.03 | 0.263 | -20.429 |
